# A Teenager Physical Fitness Evaluation Model Based on 1D-CNN with LSTM and Wearable Running PPG Recordings

**DOI:** 10.3390/bios12040202

**Published:** 2022-03-28

**Authors:** Junqi Guo, Boxin Wan, Siyu Zheng, Aohua Song, Wenshan Huang

**Affiliations:** 1 School of Artificial Intelligence, Beijing Normal University, Beijing 100875, China; guojunqi@bnu.edu.cn (J.G.); 202121081008@mail.bnu.edu.cn (A.S.); 202121081003@mail.bnu.edu.cn (W.H.); 2 Engineering Research Center of Intelligent Technology and Educational Application, Ministry of Education, Beijing 100875, China; 3 School of Electronic Information and Automation, Tianjin University of Science and Technology, Tianjin 300222, China; zhengsiyu0212@163.com

**Keywords:** teenager physical fitness monitoring, wearable bracelets, noninvasive biosensors, wireless biosensors, Photoplethysmography (PPG), Pearson correlation coefficient (PCC), deep learning, Convolutional Neural Network (CNN), Long Short-Term Memory (LSTM)

## Abstract

People attach greater importance to the physical health of teenagers because adolescence is a critical period for the healthy development of the human body. With the progress of biosensing technologies and artificial intelligence, it is feasible to apply wearable devices to continuously record teenagers’ physiological signals and make analyses based on modern advanced methods. To solve the challenge that traditional methods of monitoring teenagers’ physical fitness lack accurate computational models and in-depth data analyses, we propose a novel evaluation model for predicting the physical fitness of teenagers. First, we collected 1024 teenagers’ PPGs under the guidance of the proposed three-stage running paradigm. Next, we applied the median filter and wavelet transform to denoise the original signals and obtain HR and SpO_2_. Then, we used the Pearson correlation coefficient method to finalize the feature set, based on the extracted nine physical features. Finally, we built a 1D-CNN with LSTM model to classify teenagers’ physical fitness condition into four levels: excellent, good, medium, and poor, with an accuracy of 98.27% for boys’ physical fitness prediction, and 99.26% for girls’ physical fitness prediction. The experimental results provide evidence supporting the feasibility of predicting teenagers’ physical fitness levels by their running PPG recordings.

## 1. Introduction

People are attaching greater importance to personal health monitoring, especially in the context of the global COVID-19 pandemic background [1]. With the development of modern technologies, Artificial Intelligence (AI) is widely implemented in Healthcare 4.0 for producing early and accurate results [2]. The Internet of Things (IoT) is working as a catalyst to enhance the power of AI applications in human healthcare [3,4,5]. Increasing attention is being paid to the physical fitness of teenagers because adolescence is a crucial period of physical and health development, from the aspect of the whole life cycle [6]. The physical fitness condition of teenagers correlates not only to the happiness of their families, but also the future of the country and the nation. Researchers in the field of healthcare, psychology, and education should make a great effort to study teenagers’ physical fitness to give them a healthier and brighter future.

Referring to ‘The Second National Physical Fitness Monitoring Report’ published by the State Sports General Administration of China, the physical fitness condition of Chinese teenagers has been declining continuously since 1985 [7]. Nowadays, teenagers are spending more time on their smartphones and computers and are less likely to go out for exercise, leading to a decline in their physical health [8,9,10]. In addition to strengthening teenagers’ physical exercise, from the aspect of the teenagers themselves, governments and researchers should study the mechanisms for physical fitness evaluation to get the physical health of teenagers under control. For example, the ‘National Students’ Physical Health Standard’, published in 2014, requires all schools in China to carry out regular physical fitness tests for students of all grades, from primary school to college. Regular monitoring can indeed contribute to a better understanding of adolescent health, but it is not convenient enough.

With the rapid development of biosensors and biosensing technologies, people have more convenient and flexible means to monitor health conditions, for example, wireless and noninvasive signal collecting biosensors, such as bracelets and brain rings [11,12,13,14,15]. Smart wearable wireless biosensors, based on IoT framework, make it feasible to collect continuous physiological recordings in neural scenarios, such as sports, games, and classrooms [16,17,18,19,20]. Li Xiaoqing proposes an IoT platform for smart maternal healthcare services with wearable devices and cloud computing, and the key technologies, which can mitigate the workload of medical staff, increase work efficiency, facilitate pregnant women going to doctors, and improve the quality of obstetrical treatment [21]. Marian Ion creates a wearable flexible pressure sensor that could be integrated into a clinically approved blood pressure monitoring device for healthcare purposes [22]. In Takafumi’s research, they monitored health and diagnosed disease in the early stage with the help of a wrist flexible heart pulse sensor, integrated with a soft pump and a pneumatic balloon membrane [23]. We can apply wearable devices like smart bracelets to continuously record teenagers’ physiological signals and analyze their physical fitness condition with the aid of modern advanced methods.

There is a trend whereby the methodology of data mining transforms gradually from simple statistical means and conventional machine learning methods to deep learning algorithms. Ling Chen applied the Statistical Program for Social Sciences (SPSS) to study teenager physical health-promoting strategies in 2014 [24]. Crouter’s team applied conventional receiver operating characteristic (ROC) curves and regression analyses in 2015 to develop prediction equations for energy expenditure, to develop and validate methods for analyzing wrist accelerometer data in youth [25]. While, in recent years, the deep learning method has been much more popular with researchers for its powerful data analysis skills. According to Bolhasani reviews, deep learning is the most popular topic having a wide range of applications, such as computer vision, natural language processing, disease prediction, drug discovery, bioinformatics, biomedicine, etc. [26]. Of these applications, healthcare and medical science-related applications are dramatically on the rise. In Dang’s research, they developed an end-to-end framework which is based on physical features embedded in raw data and a 1D-CNN-LSTM model for smart structural health monitoring purposes [27]. Focusing on obesity problems in adolescents, Lee Sungchul’s team used feedforward deep learning models and CNN models to distinguish walking movements between nonobese and obese groups, at a rate of 90.5% [28]. The deep learning method can analyze the huge and complicated physical health data of teenagers more efficiently and quickly. However, at present, research on the analyses of the physical health of ordinary teenagers is still relatively lacking.

Taken together, researchers have made great efforts in the field of health monitoring of teenagers, and research methods are transforming gradually from traditional artificial statistics to computer sciences. However, experts and scholars have focused more on the healthcare of patients than on the physical development of normal adolescents. Although deep learning algorithms are growing rapidly, there are few health monitoring models for the average teenager. In order to monitor the physical health fitness condition of normal teenagers and help their teachers, families, and themselves know better about adolescents’ health, in the present research, we collected one thousand and twenty-four fourteen-year-old middle school students’ photoplethysmography signals under the guidance of our proposed three-stage running experiment paradigm. Then we applied the median filter and wavelet transform to denoise the original signals to make signals available to obtain heart rate and blood oxygen saturation. We used the Pearson correlation coefficient method to finalize the feature set based on our extracted nine physical features. Finally, we built a 1D-CNN with LSTM model to classify teenagers’ physical fitness condition into four levels: excellent, good, medium, and poor.

We organized the remainder of the paper as follows. Section 2 introduces materials and methods, including the framework system (Section 2.1), the experimental paradigm (Section 2.2), and participants’ composition (Section 2.3). We also introduce the methods of preprocessing the original physiological signals (Section 2.4), extracting key features (Section 2.5), and constructing evaluation models (Section 2.6). The finalized feature set (Section 3.1 and Section 3.2) and the evaluation metrics used to measure the performance of the proposed model (Section 3.3) are given in Section 3. Section 4 discusses the advantages and disadvantages of the proposed framework and presents the main contributions of the work. Finally, Section 5 concludes the paper.

## 2. Materials and Methods

### 2.1. System Framework

#### 2.1.1. Self-Designed Smart Bracelet System

**Figure 1:**
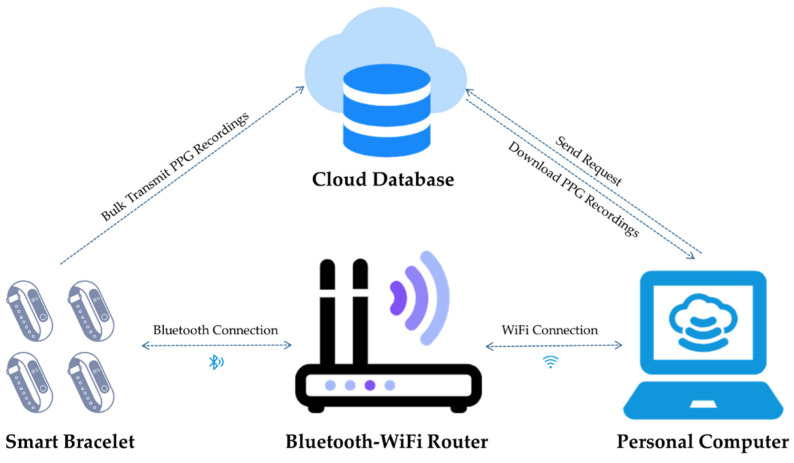
Self-designed smart bracelet system.

Our self-designed smart bracelet system is applied to achieve a bulk transmission of PPG recordings from the smart bracelets to the personal computer (PC), as displayed in Figure 1. The embedded sensors in the smart bracelets we designed collect PPG voltage value recordings 25 times per second. The effective operation of the whole system is shown below.

(1) Enable wireless connection. Bracelets keep on, and connect with, the Bluetooth-WiFi router via embedded Bluetooth modules wirelessly. Personal computers connect with the router through WiFi to ensure that the bracelets, the router, and the PC are all in the same WiFi environment.
(2) Data upload cloud. PPGs can be bulk transmitted automatically to the cloud database via Bluetooth.
(3) Gain data. If the PC sends a request to the cloud database to obtain the data stored there, we receive original signals collected by all used bracelets locally.

In this research, we first collected teenagers’ PPG recordings with the aid of smart bracelets and then applied the self-designed system to transmit the PPGs to computers for further analyses.

#### 2.1.2. Framework of the Proposed Evaluation System

**Figure 2:**
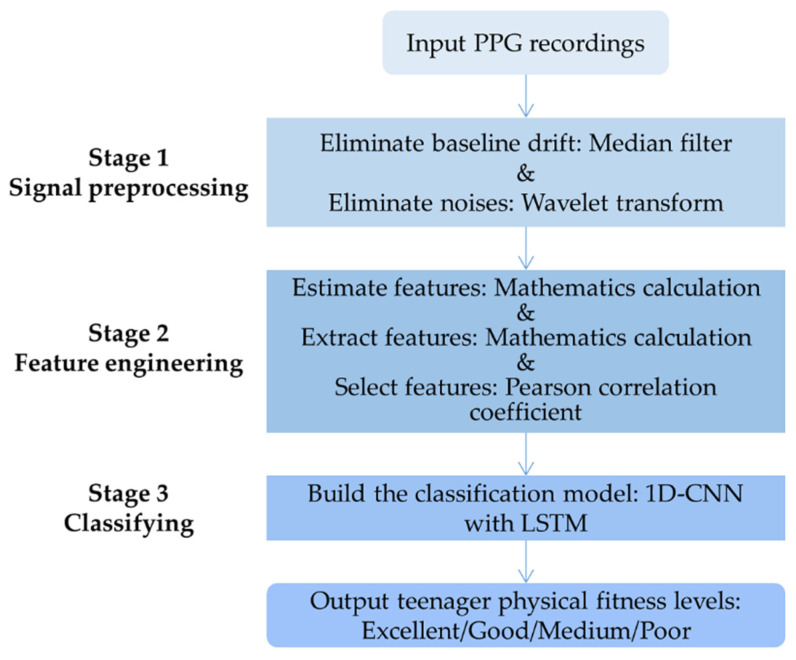
Framework of the proposed evaluation system.

To classify teenagers’ physical fitness condition into four levels, categorized as excellent, good, medium, and poor, we analyzed their physiological recordings, and we developed the framework of teenager physical fitness evaluation system shown in Figure 2. Firstly, we recorded 1024 fourteen-year-old middle school students’ PPGs under the guidance of the designed experimental paradigm. Secondly, we applied an MF-WT method to preprocess the original PPGs to ensure that the signals were clean enough for further analyses. Then, we calculated HR and SpO_2_ from the preprocessed signals and extracted nine available features. We applied PCC to finalize the feature set. Finally, a 1D-CNN with LSTM evaluation model was developed to classify the teenagers’ physical fitness condition into four levels.

### 2.2. Experimental Paradigm

The principles of the experimental paradigm to collect effective PPGs can be summarized into the following three considerations:

(1) The whole process should be based on the ‘National Students’ Physical Health Standard’ and the regular health test standard for teenage students, so that we can score students’ physical fitness condition with the aid of official principles, which will be used to guide us in labelling the running PPG features. Taking into account differences in muscle mass, respiratory development, and exercise duration between boys and girls during adolescence, the official documents state that boys run 1000 m, and girls run 800 m, so that the physical test can better monitor every person’s physical fitness. In this research, we evaluated boys’ and girls’ fitness conditions separately.
(2) Our participants were all middle school students aged 14, so it required the testing procedures to be convenient and easy, so as to avoid students being unable to wear our smart bracelets comfortably, and to ensure that we could collect available PPGs.
(3) The whole procedure ought to be scientific and complete so that we can extract as many vital physiological features as possible from the original signals.

**Figure 3:**
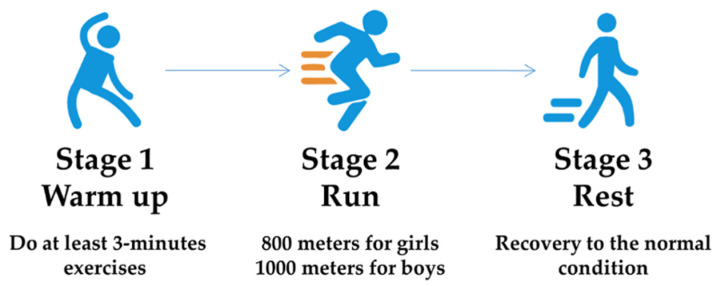
Flowchart of the experimental paradigm.

According to the three aspects above, we designed the following experimental paradigm. Volunteers wore smart bracelets and did three-minute warm-up exercises before the official run. Then they were divided into two groups based on their gender, and the boys ran 1000 m while the girls ran 800 m, respectively. One experiment allowed attendance of ten students maximum. After running, students needed to have a rest to let their rapid breathing and heartbeats slow down until they recovered to normal fitness condition. The flowchart of the experimental paradigm is shown in Figure 3.

### 2.3. Volunteer Participants

One thousand and twenty-four middle school students (576 boys and 448 girls) volunteered to participate in the teenager fitness evaluation experiment. We required all students to have adequate sleep the night before the experiment and to be in good health. This research was conducted under China’s law, and all volunteers were informed that the biosensing recordings collected would only be used for the scientific study and would not be used for other purposes. We conducted the experiment with the subjects’ consent. The collected data was processed by means of anonymity, which guaranteed that the participants’ privacy would not be divulged.

### 2.4. Signal Preprocessing

Photoplethysmography (PPG) was first proposed in 1938 by Hertzman [29]. It is a non-invasive method to detect change of blood volume in living tissues, which requires the help of photoelectric means [29]. During movement, PPGs are highly susceptible to light in the environment, electromyography noise [30], and baseline drift [31]. The last two factors seriously interfere with the accuracy of heartbeat and blood pressure monitoring, which are both involved in the original PPGs collected during the experiment.

Baseline drift occurs because of noise with a frequency below 1 Hz caused by respiration and the relative friction between the human skin surface and the PPG sensor. The PPG signal with baseline drift is the superposition of the characteristic waveform and baseline drift signal. The baseline drift signal can be eliminated by appropriate filtering [32]. The median filter (MF) is a nonlinear digital filter technology, which can be applied to eliminate noise in images or other signals [33]. The design idea of MF is to check the sampling in the input signal and judge whether it represents the signal, which can be realized with the aid of the observation window composed of odd samples. The values in the observation window are sorted, and the median in the middle of the observation window is output. Then, the earliest value is discarded, a new sample obtained, and the above calculation process repeated.

Electromyography noise generates because of muscle tremors, which is also the main reason for motion artifacts [34]. Although the action time of muscle tremors is short, it can lead to wide frequency distribution similar to white noise in the frequency domain, which will affect the reliability of PPGs. Wavelet transform (WT) is a new transform analysis method, which can analyze the localization of time or space frequency, and gradually refine the signal or function through expansion and translation operation [35]. WT can achieve time subdivision at high frequency and frequency subdivision at low frequency, and can automatically adapt to the requirements of time-frequency signal analysis. WT protects useful signal spikes and abrupt signals. It is suitable for denoising the transient signal, as well as restraining the interference of high-frequency noise. The fundamental definition formula is as follows:

(1) $$\mathrm{WT}\left(\alpha ,\tau \right)=\frac{1}{\sqrt{\alpha }}\int_{-\infty }^{\infty }f\left(t\right)*\Psi \left(\frac{t-\tau }{\alpha }\right)\mathrm{dt},$$

α means scale and controls the scaling of the wavelet function, τ means translation and controls the migration of the wavelet function. Ψ (t) is the mother wavelet function.

### 2.5. Feature Engineering

Heart rate (HR) and blood oxygen saturation (SpO_2_) are two vital indexes extracted from preprocessed PPGs. The value of HR of healthy people is between 60 to 100, and the percentage of SpO_2_ is generally more than 94% [36]. The two indexes are applied to judge many vital functions of the human body. HR is one of the most important factors for monitoring the status of human hearts. As for SpO_2_, if the blood oxygen content of the human body is insufficient, it easily causes many complications, such as headaches. Real-time monitoring of HR and SpO_2_ helps people better know their health condition.

#### 2.5.1. Feature Estimation

Direct current (DC) signals generate if no large-scale movement is in the human body because the light absorption of bones, muscles, and veins is unchanged. While alternating current (AC) signals are produced because light absorption naturally changes because of blood flow in the arteries. We applied Fast Fourier Transformation (FFT) to obtain HR from the original PPGs. We obtained the signal with a prominent amplitude near 1 Hz on the frequency spectrum. If the frequency is recorded as f, HR can be calculated as:

HR = f ∗ 60 (bpm).(2)

When we apply the photoplethysmography method to obtain SpO_2_, the skin of the human wrist is the transparent container containing hemoglobin. Both the red light with wavelength of 660 nm and the near-infrared light with wavelength of 940 nm are the incident light sources. We can measure light conduction intensity through the tissue bed to calculate SpO_2_. It provides a continuous non-invasive blood oxygen measuring instrument for the clinic. SpO_2_ and the relative light intensity of 660 nm and 940 nm on the photodetector have a linear relationship if the oxygen content in the blood changes. The calculation formula is:

(3) SpO2=a+b∗R, R=AC660DC660AC940DC940,

a and b are calibration constants:110 and 25, AC_660_ and DC_660_ mean the alternating and direct current generated in the human wrist tissue bed under red light with wavelength of 660 nm, AC_940_ and DC_940_ represent alternating and direct current generated under near-infrared light with wavelength of 940 nm.

#### 2.5.2. Feature Extraction

To extract as many features which may relate to the physical fitness levels of teenagers as possible, we defined an initial feature set, including resting heart rate, heart rate descent rate, heart rate increase rate, maximum heart rate, heart rate reserve, mean blood oxygen saturation, standard deviation of blood oxygen saturation, instant heart rate, and time duration. The definitions are shown as follows:

(1) Resting heart rate (HR_rest_) [37] refers to the number of heartbeats every minute when people are resting or in a peaceful state.
(2) Heart rate increase rate (HR_increase_) [38] refers to the rate of increase of the heart rate. Concerning that, our bracelets can collect energy consumption (EC) simultaneously while recording HR. We calculate the incremental quantity of the HR in the first 20 s of the running period divided by the incremental quantity of EC over the same time period as the HR_increase_.
(3) Maximum heart rate (HR_max_) [39] refers to the maximum value of the HR at the maximum load intensity. We can use HR_max_ to monitor teenagers’ exercise intensity.
(4) Heart rate reserve (HR_reserve_) [40] refers to the difference between HR_max_ and HR_rest_. The higher the value of HR_reserve_ is, the better the cardiopulmonary function is.
(5) Mean blood oxygen saturation (SpO_2mean_), SpO_2_ shows significant changes for neither the average person nor professional athletes under normal conditions, but it will apparently decrease when there is a quantity load of exercise. To extract as many adequate features as possible, which may be correlated with the teenagers’ physical fitness levels, we add SpO_2mean_ into our initial feature set.
(6) Standard deviation of blood oxygen saturation (SpO_2SD_) [41], SpO_2SD_ shows little variation. However, we also add this feature into the original set to verify whether it makes sense to monitor teenagers’ physical fitness condition.
(7) Time duration (TD) refers to the minus value between the time of finishing running (T_end_)and the time of starting running (T_start_). According to the regular health test standard for teenage students, TD decides the results of the running process. The shorter TD is, the better the score is. Referring to TD, we can distribute the teenagers’ fitness levels into four degrees: excellent, good, medium, and poor.
(8) Instant heart rate (HR_instant_) [42] refers to the immediate HR after exercise. Zhou’s research finds that HR_instant_ has a strong negative correlation with cardiopulmonary functions, which shows that HR_instant_ may have a strong correspondence with predicting teenagers’ fitness levels.
(9) Heart rate descent rate (HR_descent_) [43] refers to the rate of recovery of heart rate after exercise, especially the minute immediately after exercise. The faster the HR drops, the better the cardiopulmonary function may be.

The definition formulae of the extracted features are shown in Table 1.

#### 2.5.3. Feature Selection

We extracted nine features that may correlate with levels of teenagers’ physical fitness. However, we made a first-round selection among these features to make sure that the selected features were key features, really contributing to evaluating physical fitness levels. The principles used to choose key features were as follows:

(1) They had a strong correlation with physical fitness levels. For example, we found SpO_2mean_ and SpO_2SD_ do not have apparent changes, so we are not sure whether these two features can evaluate teenagers’ fitness condition. It was necessary to take some measures to remove useless features.
(2) They did not have redundancy information between each other. Take SpO_2mean_ and SpO_2SD_ for example, they are both extracted from SpO_2_, so there may be redundancy information between them. We needed to find a way to remove the redundant features to make the selected dataset as clean as possible.

Based on the two principles above, we needed to analyze the correlation among extracted features to finalize the feature set. The final purpose of our research was to classify the teenagers’ physical fitness levels based on the PPGs, so it was necessary that we considered the correlated relationship between the feature and the fitness level when we made correlation analyses.

In the field of natural science, the Pearson correlation coefficient (PCC) is widely applied to measure correlation between two variables, and its value ranges from −1 to 1 [44]. It evolved from a similar, but slightly different, idea put forward by Francis Galton, in the 1880s.

PCC between two variables is defined as the quotient of covariance and standard deviation:

(13) $$\rho_{X,Y}=\frac{\mathrm{cov}(X,Y)}{\sigma_{X}\sigma_{Y}}=\frac{E\left[\left(X-\mu_{X}\right)\left(Y-\mu_{Y}\right)\right]}{\sigma_{X}\sigma_{Y}}.$$

The above formula defines the overall correlation coefficient, which is usually represented by ρ. $\varrho_{X}$ and $\varrho_{Y}$ to represent the standard deviation of two variables, respectively. Cov(X,Y) represents the covariance of two variables.

PCC can also be obtained by estimating the covariance and standard deviation of the sample, which is commonly represented by r:

(14) $$r=\frac{\sum_{i=1}^{n}\left(X_{i}-\bar{X}\right)\left(Y_{i}-\bar{Y}\right)}{\sqrt{\sum_{i=1}^{n}{\left(X_{i}-\bar{X}\right)}^{2}}\sqrt{\sum_{i=1}^{n}{\left(Y_{i}-\bar{Y}\right)}^{2}}}.$$

The value of PCC varies from −1 to 1. Value ‘1’ means that X and Y can be well described by linear equation. All data points fall on a straight line, and Y increases with the increase of X. Value ‘−1’ means that all data points fall on a straight line, while Y decreases with increase of X. Value ‘0’ means that the two variables have no relationship. Table 2 shows the relationship between the absolute value of PCC and correlation degrees.

### 2.6. A Deep Learning Method: 1D-CNN with LSTM

Professor Lecun first proposed the LeNet model based on Convolutional Neural Network (CNN) to solve the visual task of handwritten numeral recognition in 1998. In recent years, researchers have applied CNN and other CNN-based variant models to image processing, object detection, signal processing, and so on [45]. Considering the multi-dimensions of the features, and the computing and dimension reduction processing ability of deep learning algorithms, we applied 1D-CNN to build the evaluation model. A fundamental CNN is mainly composed of input layer, convolution layer, pooling layer, full connection layer, and activation layer. The function of each layer is briefly described as follows.

(1) The input layer is a layer for the network to receive input samples. Its shape is consistent with that of the input samples. It serves to receive and transmit data for the network behind this layer.
(2) The function of the convolution layer is to obtain features of input data through convolution operation, extract multiple features by using convolution kernels of different scales, and increase the number of effective features. The number of convolution kernels can change the depth of the input matrix. The convolution operation is such that the convolution kernel traverses the input matrix and convolutes with the elements at the corresponding position in the input matrix. A new output matrix starts from the operation result.
(3) The pooling layer reduces the length and width of the matrix, which can reduce the size of the matrix, as well as the number of network parameters. The pooling layer usually discards the part with less information and retains its vital information, which not only speeds up calculation efficiency, but also ensures accuracy.
(4) The full connection layer is a hierarchy that connects all nodes between previous features and final output.
(5) The Softmax layer maps the input to real numbers between 0 and 1 and then normalizes these real numbers to ensure that the sum is 1; that is, to ensure the probability of mapping the input to the corresponding category. Through the transformation of the Softmax layer, the output of the previous layer is transformed into the probability that the samples belong to each class, so that the network can complete the multi-classification task.

Long Short-Term Memory (LSTM) was first proposed by Hochreiter and Schmidhuber in 1997 [46]. The original design intention was to solve the problem of long-term dependence in neural networks and make remembering long-term information the default behavior of neural networks, rather than requiring much effort to learn [47]. LSTM controls discarding or adding information through a ‘gate’ to realize the function of forgetting or remembering. A ‘gate’ is a structure that allows information to pass selectively, which is composed of a sigmoid function and a dot multiplication operation. The output value of the sigmoid function is between 0 and 1. ‘0’ represents complete discarding, while ‘1’ represents complete passing. An LSTM unit has three such gates: forget gate, input gate, and output gate.

Recurrent neural network (RNN) is suitable for processing time-series data, like one-channel physiological recordings. However, the original RNN has the problem of gradient disappearance, or gradient explosion. LSTM and layered RNN are all solutions to this problem. The essence of LSTM is to introduce the concept of cell state. Unlike RNN, which only considers the recent state, the cell state of LSTM will determine which states should be left and which should be forgotten [47]. Basic RNN can deal with some short-term dependence, but it cannot deal with long-term dependence, while LSTM has this capability.

Considering that the physiological characteristics we screen out are one-dimensional time-allowed sequences, we propose a 1D-CNN with an LSTM evaluation model to achieve the classified task. In the proposed model, the Conv1D layer refers to one-dimensional convolution calculation, which is usually used to process one-dimensional data, such as physiological signals. ReLU (Rectified Linear Unit), also known as modified linear unit, is an activation function that refers to the nonlinear functions represented by slope function and its variants. Batch normalization is a method to unify the scattered data, and also to optimize the neural network. The dropout layer prevents overfitting and improves the generalization ability of the model. The dense layer can aggregate network information for classification or other purposes.

## 3. Experiment Results

### 3.1. Signal Preprocessing Results

**Figure 4:**
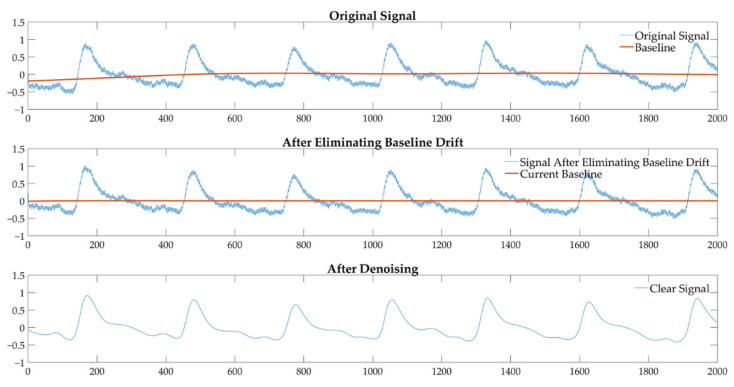
An example of simulated physiological recordings using the preprocessing methods.

We applied MF and WT to preprocess the original PPGs to ensure the signals were clean enough for feature estimation. Figure 4 is an example of simulated physiological recordings with the preprocessing methods. The preprocessed signals with non-baseline draft and little noise welcomed further analysis.

### 3.2. Feature Engineering Results

**Figure 5:**
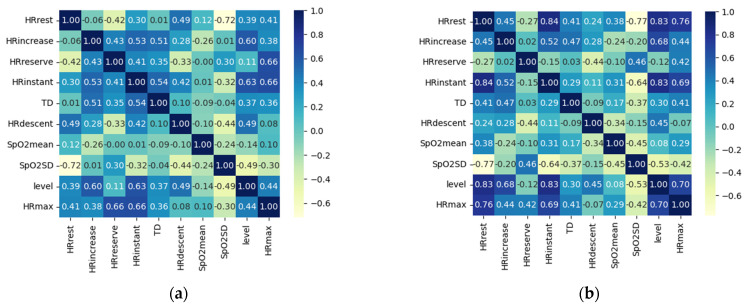
PCC among all features. (**a**) PCC among features extracted from boys’ recordings; (**b**) PCC among features extracted from girls’ recordings.

Values and correlated degrees of the Pearson correlation coefficient between teenagers’ physiological features and their physical fitness levels are displayed in Table 3 and Table 4. The values of PCC among physiological features are shown in Figure 5, in which image (a) on the left is for boys, and image (b) on the right is for girls.

As regards boys’ features, there is no very strong correlation between physiological features and fitness levels. But HR_increase_ (PCC = 0.598), HR_max_ (PCC = 0.440), SpO_2SD_ (PCC = −0.493), HR_descent_ (PCC = 0.492), and HR_instant_ (PCC = 0.625) show a moderate or strong correlation. We added those features to our selected feature set. HR_rest_ and TD’s PCC is between 0.2 and 0.4, representing a weak relationship between them and fitness levels. From image (a) in Figure 5, we find that HR_rest_ has a strong correlation with SpO_2SD_ (PCC = −0.72). SpO_2SD_ is already in the selected feature set, so we eliminated HR_rest_. TD was the only scoring criteria for scoring students’ running in middle schools, although the PCC between TD and fitness levels is 0.370, we chose to persist with TD. HR_reserve_ (PCC = 0.113) and SpO_2mean_ (PCC = −0.136) have an irrelevant correlation to fitness levels, so we removed them directly.

As regards girls’ features, HR_rest_ (PCC = 0.826) and HR_instant_ (PCC = 0.830) have a very strong correlation with fitness levels. We added these two features in the final feature set. We removed HR_reserve_ and SpO_2mean_ directly because their values are −0.119 and 0.082, respectively. The PCC between TD and girls’ physical fitness levels is 0.299, lower than 0.4, but for the same reason as regards the boys, we kept TD in the final selected feature set. The PCC between HR_max_ and girls’ physical fitness levels is 0.699, representing a strong correlation, but HR_max_ has a strong relationship with HR_rest_ (PCC = 0.76) and HR_instant_ (PCC = 0.69) referring to image (b) in Figure 5. We considered that there was redundant information among HR_max_, HR_rest_, and HR_instant_, so we removed HR_max_ from the final feature set. SpO_2SD_ (PCC = −0.529) and HR_descent_ (PCC = 0.450) show a moderate relationship, but HR_descent_ shows a weak correlation with other features. We cannot delete HR_instant_ casually. SpO_2SD_ has a strong correlation with HR_rest_ (PCC = −0.77) and HR_instant_ (PCC = −0.64), which were already chosen in the final set, so we removed SpO_2SD_.

Table 5 shows the final selected feature sets for boys and girls. We chose six features for the boys’ feature set and 5 features for the girls’ feature set. The final selected feature set will be used to develop our teenager physical fitness evaluation model.

### 3.3. Evaluation Model Performance

We applied the proposed 1D-CNN with LSTM model to evaluate teenagers’ physical fitness condition. When developing the proposed model, we divided the data set in a ratio of seven to three and reduced the learning rate to the original 0.5 every 100 epochs. After several rounds of frame adjustment, we constructed the final framework described in Table 6 because of its best performance.

**Figure 6:**
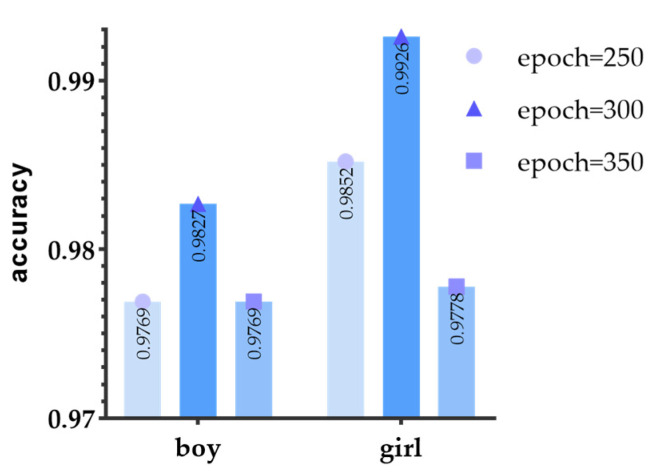
Different accuracies for different epochs. The dark blue column with a triangle performs the best.

To better measure the performance of the model, we recorded accuracy for the whole model, as well as precision, recall, and f1-score for every class with different epochs (epoch = 250, 300, and 350). Table 7 and Figure 6 record the evaluation indexes for teenagers’ physical fitness levels. We found that when the training epoch is 300, accuracy reaches the highest, the values of boys’ and girls’ physical fitness evaluation accuracy are 98.27% and 99.26%, respectively. From the detailed evaluation indexes for every class, we found two edge classifications (excellent and poor) get 100% accuracy for every epoch, for both boys and girls. While the accuracy for classifications of two intermediate parts (good and medium, especially medium) hardly achieve 100% prediction accuracy.

**Figure 7:**
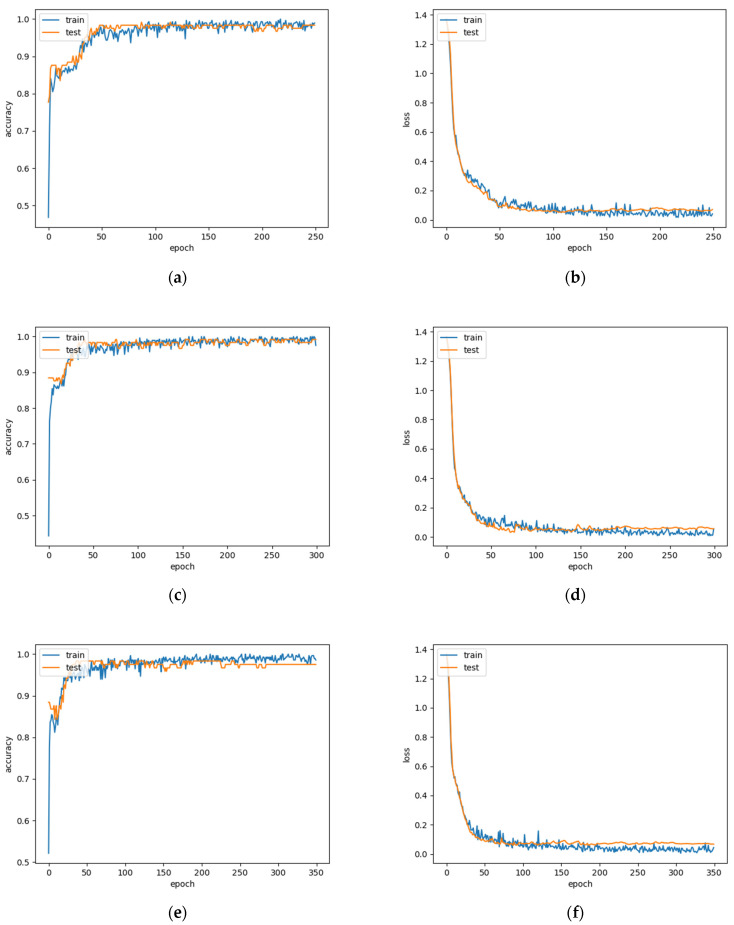
Accuracy and loss for boys’ physical fitness level evaluation. (**a**,**b**) Accuracy and loss when epoch is 250; (**c**,**d**) Accuracy and loss when epoch is 300; (**e**,**f**) Accuracy and loss when epoch is 350.

**Figure 8:**
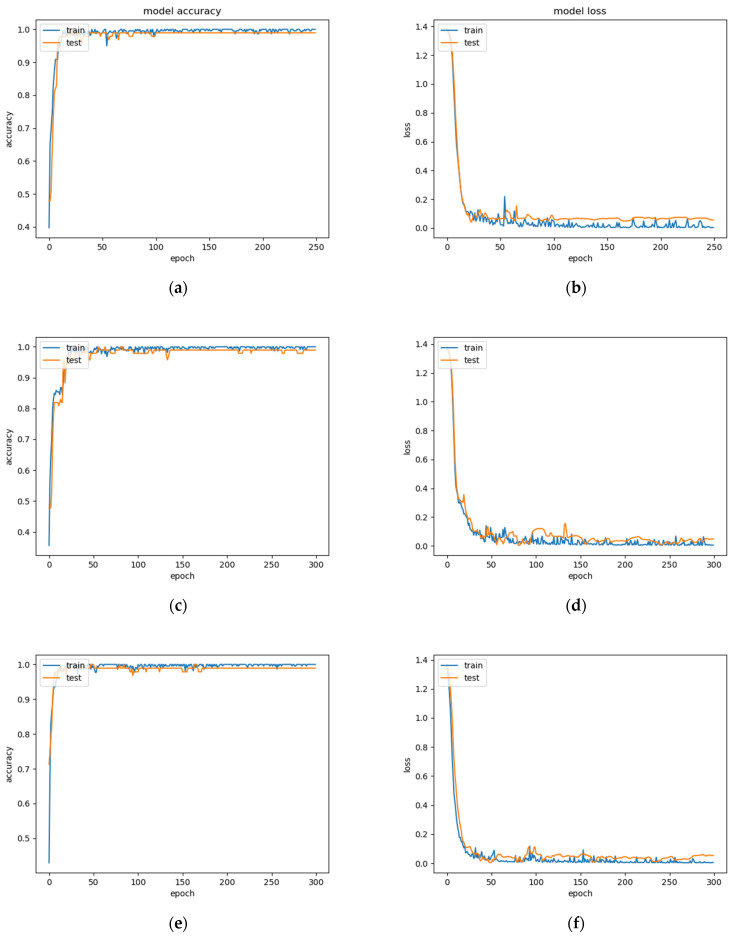
Accuracy and loss for girls’ physical fitness level evaluation. (**a**,**b**) Accuracy and loss when epoch is 250; (**c**,**d**) Accuracy and loss when epoch is 300; (**e**,**f**) Accuracy and loss when epoch is 350.

We recorded the changes of accuracy and loss during different epoch training for both boys and girls in Figure 7 and Figure 8. Decreasing loss value proves that it is converging, and increasing accuracy value proves that its accuracy is improving.

**Figure 9:**
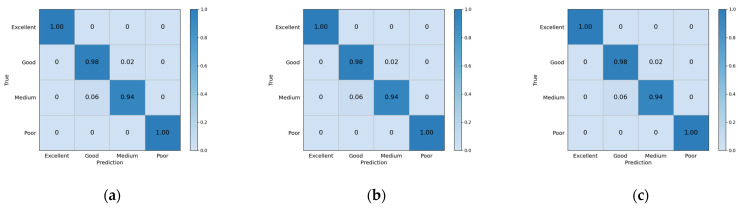
Confusion matrix for boys’ physical fitness level evaluation. (**a**) Confusion matrix when epoch is 250; (**b**) Confusion matrix when epoch is 300; (**c**) Confusion matrix when epoch is 350.

**Figure 10:**
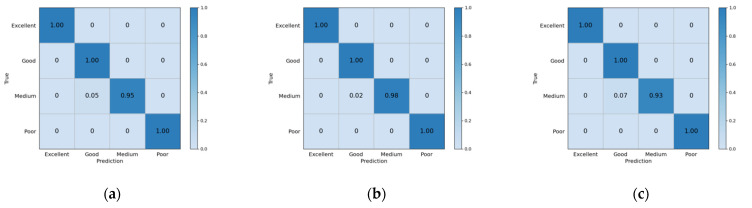
Confusion matrix for girls’ physical fitness level evaluation. (**a**) Confusion matrix when epoch is 250; (**b**) Confusion matrix when epoch is 300; (**c**) Confusion matrix when epoch is 350.

From Figure 9 and Figure 10, we can more clearly find that accuracy in classifying two intermediate parts (good and medium, especially medium) hardly achieves 100% prediction accuracy. The accuracy rates of excellent and poor are pretty high. We consider the middle two levels: good and medium, to be excessive, and hard to classify, while excellent and poor are more widely separated and easily distinguishable.

## 4. Discussions

In the present research, we explored predictability of the running PPGs for the physical fitness levels of teenagers. The wrist-recorded PPGs were found to be an effective indicator of teenagers’ physical fitness condition, with better results for predicting excellent and poor levels. Intermediate health levels are a bit harder to assess. The experiment results indicate that people can monitor physical fitness condition of adolescents by analyzing their physiological recordings with modern artificial intelligence methods.

Through mathematical calculation, MF-WT denoising, and PCC value analyses, we finalized the physiological feature set for boys and girls, respectively. Considering the one-dimensional nature, and time correlation, of physiological signals, computing ability, and dimension reduction processing ability of deep learning, we applied 1D-CNN with LSTM to achieve the classified task and got 98.27% (for boys) and 99.26% (for girls) as the final prediction results. Notably, whereas, ideally, it is expected to collect physiological recordings from all items of the student physical fitness test, on the guidance of official documents published by the government, for the prediction of health condition, our data were measured merely by a typical running test. Although cardiopulmonary function can be detected through long-distance running, and running is easier to monitor than sports such as those requiring sitting forward flexion, we admit that if combined with more physical tests, the results can be more accurate. Furthermore, the accuracy of the evaluation model, based on the current method, has reached the commercial level, but if the signals collected from other sports are added in the future, the accuracy will not be known.

In summary, the contributions of this paper are summarized as follows:

(1) We have pioneered the exploration of the relationship between physiological recordings of teenagers with their physical fitness levels and proposed that some key features could be effectively used to predict fitness levels.
(2) We have proposed an assessment model based on an optimized 1D-CNN with LSTM to predict the outcome of the running physical test. The proposed model could also be used for other predictive tasks based on biosensing recordings.
(3) The experimental results provide evidence supporting the feasibility of predicting teenagers’ physical fitness levels by their biosensing recordings.

## 5. Conclusions

In this research, we have proposed a 1D-CNN with LSTM based evaluation model to assess the physical fitness condition of teenagers with the aid of our self-designed wearable smart bracelet system. Under the guidance of the designed experimental paradigm, we collected one thousand and twenty-four fourteen-year-old middle school students’ PPGs and applied median filter and wavelet transform to make the original physiological recordings clean enough for further mathematical calculating of heart rate (HR) and blood oxygen saturation (SpO_2_). Then we calculated nine features based on HR and SpO_2_ extracted in different experimental procedures and used the Pearson correlation coefficient method to finalize the feature set. Finally, we constructed a deep learning model, 1D-CNN with LSTM, to classify teenagers’ physical fitness condition into four levels: excellent, good, medium, and poor, with an accuracy of 98.27% for boys, and 99.26% for girls.

Our research demonstrates the possibility and feasibility of applying deep learning methods to analyze teenagers’ physical fitness based on PPG recordings, and fills the gap of health monitoring for ordinary teenagers. Extending our knowledge on the healthcare of teenagers may contribute to improving routine physical assessments and arousing widespread concern in society. With the rapid progress of biosensing technologies and artificial intelligence, health monitoring for teenagers’ physical fitness will achieve faster development based on existing methods.

## Figures and Tables

**Table 1:** Definition formulae of the 9 features.

Measurement Period	Feature	Formulae	
3-min warm-up exercise	Resting heart rate (HR_rest_)	${\mathrm{HR}}_{\mathrm{rest}}=\frac{1}{3*60}\overset{T_{\mathrm{start}+3*60}}{\underset{{t=T}_{\mathrm{start}}}{\sum }}{\mathrm{HR}}_{t}$	(4)
Running	Heart rate increase rate (HR_increase_)	${\mathrm{HR}}_{\mathrm{increase}}=\frac{{\mathrm{HR}}_{T_{\mathrm{start}}+20}-{\mathrm{HR}}_{T_{\mathrm{start}}}}{{\mathrm{EC}}_{T_{\mathrm{start}+20}}-{\mathrm{EC}}_{T_{\mathrm{start}}}}$	(5)
	Maximum heart rate (HR_max_)	${\mathrm{HR}}_{\max}=\max({\mathrm{HR}}_{t}),t=T_{\mathrm{start}}\ldots T_{\mathrm{end}}$	(6)
	Heart rate reserve (HR_reserve_)	${\mathrm{HR}}_{\mathrm{reserve}}={\mathrm{HR}}_{\max}-{\mathrm{HR}}_{\mathrm{rest}}$	(7)
	Mean blood oxygen saturation (SpO_2mean_)	${\mathrm{SpO}}_{2\mathrm{mean}}=\frac{\sum_{{t=T}_{\mathrm{start}}}^{T_{\mathrm{end}}}{\left({\mathrm{SpO}}_{2}\right)}_{t}}{T_{\mathrm{end}}-T_{\mathrm{start}}}$	(8)
	Standard deviation of blood oxygen saturation (SpO_2SD_)	${\mathrm{SpO}}_{2\mathrm{SD}}=\sqrt{\frac{1}{T_{\mathrm{end}}-T_{\mathrm{start}}}\overset{T_{\mathrm{end}}-T_{\mathrm{start}}}{\underset{t=T_{\mathrm{start}}}{\sum }}{\left({\mathrm{HR}}_{t}-{\mathrm{SpO}}_{2\mathrm{mean}}\right)}^{2}}$	(9)
	Time duration (TD)	$\mathrm{TD}=T_{\mathrm{end}}-T_{\mathrm{start}}$	(10)
Recovery	Heart rate descent rate (HR_decent_)	${\mathrm{HR}}_{\mathrm{descent}}=\frac{\sum_{t=T_{\mathrm{end}}}^{T_{\mathrm{end}+10}}{\mathrm{HR}}_{t}-\sum_{t=T_{\mathrm{end}+110}}^{T_{\mathrm{end}+120}}{\mathrm{HR}}_{t}}{10}$	(11)
	Instant heart rate (HR_instant_)	${\mathrm{HR}}_{\mathrm{instant}}={\mathrm{HR}}_{T_{\mathrm{end}}}$	(12)

**Table 2:** The relationship between PCC and correlation degrees.

**PCC**	0.8~1.0	0.6~0.8	0.6~0.4	0.2~0.4	0.0~0.2
**Relationship**	Very strong	Strong	Moderate	Weak	Irrelevant

**Table 3:** Values of the Pearson correlation coefficient between teenagers’ initial features and their fitness levels.

Feature		HR_rest_	HR_increase_	HR_max_	HR_reserve_	SpO_2mean_	SpO_2SD_	TD	HR_descent_	HR_instant_
**PCC**	Boy	0.392	0.598	0.440	0.113	−0.136	−0.493	0.370	0.492	0.625
	Girl	0.826	0.676	0.699	−0.119	0.082	−0.529	0.299	0.450	0.830

**Table 4:** Features distributed by degrees of correlation to physical fitness levels.

Correlation		Very Strong (PCC: 0.8~1.0)	Strong (PCC: 0.6~0.8)	Moderate (PCC: 0.4~0.6)	Weak (PCC: 0.2~0.4)	Irrelevant (PCC: 0.0~0.2)
**Gender**	Boy	None	HR_instant_	HR_max_	HR_rest_	HR_reserve_
				SpO_2SD_		
				HR_descent_	TD	SpO_2mean_
				HR_increase_		
	Girl	HR_rest_	HR_increase_	SpO_2SD_	TD	HR_reserve_
		HR_instant_	HR_max_	HR_descent_		SpO_2mean_

**Table 5:** Final selected feature set. ‘√’ represents that we added this feature to the final feature set, ‘×’ means the opposite.

Feature		HR_rest_	HR_increase_	HR_max_	HR_reserve_	SpO_2mean_	SpO_2SD_	TD	HR_descent_	HR_instant_
**Gender**	Boy	×	√	√	×	×	√	√	√	√
	Girl	√	√	×	×	×	×	√	√	√

**Table 6:** The framework of teenagers’ physical fitness evaluation models.

Model Framework for Boys			Model Framework for Girls		
Layer Type	Output Shape	Parameters	Layer Type	Output Shape	Parameters
Conv1D	(None, 4, 64)	192	Conv1D	(None, 4, 64)	192
ReLU	(None, 4, 64)	0	ReLU	(None, 4, 64)	0
Conv1D	(None, 2, 64)	12352	Conv1D	(None, 2, 64)	12352
Conv1D	(None, 1, 64)	4160	Conv1D	(None, 1, 64)	4160
Batch normalization	(None, 1, 64)	256	Batch normalization	(None, 1, 64)	256
Dropout	(None, 1, 64)	0	Dropout	(None, 1, 64)	0
Conv1D	(None, 1, 32)	2080	Conv1D	(None, 1, 64)	4160
Conv1D	(None, 1, 32)	1056	Conv1D	(None, 1, 64)	4160
Batch normalization	(None, 1, 32)	128	Batch normalization	(None, 1, 64)	256
LSTM	(None, 1, 32)	8320	LSTM	(None, 1, 64)	33024
LSTM	(None, 32)	8320	LSTM	(None, 32)	12416
Dense	(None, 4)	132	Dropout	(None, 32)	0
			Dense	(None, 4)	132

**Table 7:** Evaluation indexes for teenagers’ physical fitness levels.

Gender	Epoch	Accuracy	Classes	Evaluation Indexes		
				Precision	Recall	F1-Score
Boy	250	0.9769	Excellent	1.00	1.00	1.00
			Good	0.99	0.98	0.98
			Medium	0.84	0.94	0.89
			Poor	1.00	1.00	1.00
	300	0.9827 ^1^	Excellent	1.00	1.00	1.00
			Good	0.99	0.98	0.99
			Medium	0.89	0.94	0.91
			Poor	1.00	1.00	1.00
	350	0.9769	Excellent	1.00	1.00	1.00
			Good	0.99	0.98	0.98
			Medium	0.84	0.94	0.89
			Poor	1.00	1.00	1.00
Girl	250	0.9852	Excellent	1.00	1.00	1.00
			Good	0.95	1.00	0.97
			Medium	1.00	0.93	0.96
			Poor	1.00	1.00	1.00
	300	0.9926 ^2^	Excellent	1.00	1.00	1.00
			Good	0.98	1.00	0.99
			Medium	1.00	0.98	0.98
			Poor	1.00	1.00	1.00
	350	0.9778	Excellent	1.00	1.00	1.00
			Good	0.95	1.00	0.97
			Medium	1.00	0.93	0.96
			Poor	1.00	1.00	1.00

^1,2^ The best accuracy for gender.

## Data Availability

The datasets within this research are available from the corresponding author on reasonable request.
